# Physical Activity Recommendations for Health and Beyond in Currently Inactive Populations

**DOI:** 10.3390/ijerph15051042

**Published:** 2018-05-22

**Authors:** Eszter Füzéki, Winfried Banzer

**Affiliations:** Department of Sports Medicine, Goethe University Frankfurt, 60487 Frankfurt, Germany; banzer@sport.uni-frankfurt.de

**Keywords:** physical activity, physical activity promotion, health, physical activity recommendations

## Abstract

Widespread persistent inactivity makes continued efforts in physical activity promotion a persistent challenge. The precise content of physical activity recommendations is not broadly known, and there are concerns that the general messaging of the guidelines, including the recommendations to perform at least 150 min of at least moderate intensity physical activity per week might seem unattainable for and even actually discourage currently inactive people. Here we show that there are a myriad of ways of being physically active, and provide (in part) out-of-the-box examples of evidence based, pragmatic, easily accessible physical activity regimes below 150 min and/or with lower than moderate intensity that yield meaningful health benefits for currently inactive people.

## 1. Introduction

The health benefits of physical activity (PA) are unquestionable. The recognition of the importance of physical inactivity as a risk factor for chronic diseases and premature mortality has led to the development of public health-oriented PA guidelines by the World Health Organization (WHO) and an increasing number of national governments worldwide [1]. PA guidelines are evidence-based documents that inform on the form, volume, intensity and frequency of PA required to maintain and promote health [2]. The existence of national recommendations is regarded as a benchmark in comprehensive national PA promotion strategies [1]. The development of national guidelines can mobilize and unite relevant stakeholders in the field and provide visibility for the cause, strengthening the position of health-related PA on the public health agenda [1].

The dose-response relationship between PA and most health outcomes is such that the highest relative health benefits occur when moving from complete inactivity to some activity, even if this volume remains well below the currently recommended amount of 150 min per week [3,4,5]. Some national PA guidelines [6], but not the WHO, recommend any level of PA, also below 150 min. A number of authors are still concerned and have voiced criticism about the current emphasis of 150 min PA as the minimum amount or the goal [7,8,9,10,11].

The practical implication of the fact that PA below the level of 150 min conveys meaningful health benefits is that public health efforts should focus on getting inactive people to do at least some physical activity. Rather than defining PA goals at the population level in terms of meeting current guidelines, as e.g., the World Health Organisation’s Global action plan for the prevention and control of non-communicable diseases 2013–2020 does, small incremental increases in PA should build the core of public health messages [7]. One example for this approach is the “Active Guide” in Japan, which advocates the adding of 10 min of PA on top of current levels of activity [12].

PA guidelines are an important benchmark as a policy measure but are not point-by-point manuals; they rather offer general guidance in terms of type, intensity, duration, and frequency of activity [2]. For this reason, providing pragmatic how-tos and supporting messages are required to facilitate PA uptake [2]. The need for simple, easy, affordable and time-conscious PA forms is also underlined by the often-cited barriers to regular PA, such as access difficulties, affordability, ref. [13] lack of time and facilities [14].

The aim of this narrative review is to provide evidence based, easy to implement, “out of the box” examples for PA interventions that have not received much interest so far, and are also below the recommended volume or intensity in the previously untrained general population. Taking the public health perspective, we focus on low burden PA regimes in terms of time, access, costs or skills beyond activity forms of active travel that are being often promoted such as walking and cycling. Furthermore, we focus on efficacy, i.e., whether these types of activities yield health benefits, rather than on effectiveness. In other words, our main interest is, whether these interventions are able to beneficially influence relevant health markers. If yes, further studies could test whether the promotion of such activities might lead to an increased PA uptake in the currently inactive. Wherever available, we cite evidence based meta-analyses, systematic reviews and randomized controlled trials.

## 2. Take It Easy

The evidence for the benefits of PA with moderate to vigorous intensity (MVPA) over a wide range of health outcomes is very strong; accordingly, this is what is being recommended by national governments, public health institutions and authorities [6,15]. Current recommendations were developed with a heavy reliance on epidemiological data, based on self-reported PA [16]. One known limitation of self-report instruments is that they poorly capture low or light intensity PA (LIPA) [17]. As a consequence, current recommendations include only activities in the moderate to vigorous intensity range. The absence of low intensity activities in current guidelines reflects more an uncertainty about possible health benefits, rather than a negative recommendation in light of a proven lack of effectiveness [16]. Recent technological development now allows the wider use of motion sensors capable of also registering LIPA in large, representative cohort studies [16]. Notably starting with the 2003/2004 wave, the National Health and Nutrition Examination Survey (NHANES) now assesses PA using accelerometers. Based on accelerometer data of the 2003/2004 and the 2005/2006 waves, we found in our recent systematic review that beneficial associations exist in a cross-sectional analysis between LIPA and obesity, as well as markers of lipid and glucose metabolism [18]. Longitudinal studies also showed a positive association between LIPA and mortality [18].

These observational data are supported at least in certain populations by experimental evidence as well. A current meta-analysis of controlled clinical trials in sedentary but apparently healthy older adults found strong evidence for a dose–response relationship between exercise intensity and improvements in cardiorespiratory fitness (CRF), with improvements starting already at the low-moderate intensity range (35–50% heart rate reserve, HRR) [19]. LIPA can also beneficially influence cardiometabolic outcomes, such as reduced postprandial glycemia [20,21], high density cholesterol [22], markers of insulin sensitivity, total and low density cholesterol concentrations [23].

Traditional resistance training recommendations suggest an intensity of 60–70% 1 repetition maximum (1 RM) in novice exercisers to improve strength [24]. Meta-analytic data show a dose-response relationship, with greater loads yielding greater increases in strength [25]. Compared to no exercise, however, loads below 60% 1 RM can also induce significant strength gains in older healthy adults [25]. Besides load, resistance training has a number of variables, such as velocity of movement, repetitions, sets, and rest periods, the manipulation of which might influence outcomes. Performing resistance training at slow velocity and without rest between repetitions has been shown to increase strength with loads as low as 30% 1RM in elderly participants [26]. Also the increase in muscle strength and size in young men exercising at 50% 1 RM at slow pace and short rest was similar to increases after “traditional” training at 80% 1 RM at normal velocity [27].

Since performing more repetitions with lower loads has been shown to be perceived as less difficult than fewer repetitions with heavier loads [28], and higher adherence and higher activity volume were achieved when intensity levels were lower [29], promoting LIPA might be promising. Taken together it seems reasonable to recommend PA of any intensity to people who are currently inactive [30].

## 3. Step It up

Besides walking and cycling, stair walking is another daily life activity that can double as exercise. The intensity of ascending and descending stairs is 9.6 and 4.8 metabolic equivalent of task (MET) respectively, i.e., stair climbing is a vigorous-moderate intensity activity [31]. Stairs can be found in a number of settings, such as in public spaces, in malls, at work places and in private homes, and they thus provide an easily available opportunity to perform PA free of charge.

Stair climbing interventions have been shown to enhance important health outcomes. A 7-week randomized controlled program in previously sedentary young women improved exercise economy and lipid profiles [32]. Participants performed stair climbing 5 times a week, and increased the volume of activity gradually, reaching about 13.5 min per session during week 6 and 7, totaling just a little over an hour per week [32]. In another randomized controlled workplace intervention office workers were encouraged to do daily stair climbing and descending workouts for 10 min per day [33]. About 83% of participants accomplished at least 3 sessions per week, i.e., effects were achieved with 30 min of activity per week. CRF increased and blood pressure decreased in the intervention group, with more pronounced improvements (10% CRF improvement) in those with low CRF at the baseline [33]. With only 20% of the study participants stating that they would definitely not continue stair walking after the end of the research project, such interventions might be considered as an alternative to “classical” exercise [33]. Bean and colleagues compared a traditional walking (3 times 45 min per week) and a weighted stair climbing (3 times 10 min per week) program in mobility limited elderly people [34]. Both programs improved submaximal aerobic performance to the same extent, and stair walking yielded significant improvements in leg press power and functional outcomes in a sub-cohort with moderate mobility limitations as measured per the Short Physical Performance Battery [34].

## 4. Alternate

Interval training, i.e., alternating short periods of higher intensity and low intensity (or rest) periods is a well-established training method in elite sports. There are a large variety of protocols, but intensity in high intensity interval training (HIIT) is typically set near maximal (≥85% VO2max, ≥85% HRR, ≥90% HRmax) [35,36]. More recently, HIIT has also gained interest in the general and even chronically ill populations [36,37,38].

### 4.1. Interval Walking

The concept of interval walking was developed in Japan for middle aged and older inactive adults. Participants are instructed to perform 5 or more series of walking consisting of alternating 3 min at ≥70% VO2peak and 3 min at 40% VO2peak on four or more days a week [39]. Thus, the intensity during the “intensive” phase is still considerably lower than in typical HIIT protocols. In the Japanese study, accelerometers measured walking intensity and provided audio signals to indicate when participants need to change intensity [39]. At 5 months the interval but not continuous walking increased the VO2peak by ~10%, and the knee extension and flexion force by 13% and 17%, respectively [39]. Systolic and diastolic pressures decreased by ~9 and ~5 mmHg respectively [39]. Long-term results suggest high adherence rates with sustained improvement in lifestyle-related disease score and 12% increase in VO2peak [40]. With the development of the “e-Health Promotion System”, it is now possible for participants to train on their own and receive regular feedback on their achievements [41].

The interval walking method has been implemented in various other countries [42] and in chronic populations as well [43]. Since it has been shown to improve physical fitness, body composition, and glycemic control more than continuous walking in diabetic patients [43], it has then been rolled out further. With the newly developed and validated InterWalk App available free of charge, it is now a part of a technology-driven public health approach for diabetic patients in Denmark [44,45].

### 4.2. Swimming

Most interval training studies use walking/running/sprinting or cycling, but this training method does not have to be limited to these activities. Swimming is a whole-body exercise that is also suitable for overweight and obese individuals, for whom joint loading activities such as walking or jogging might not be feasible. Middle-aged, inactive women with mild to moderate arterial hypertension were randomized to the high-intensity intermittent (6–10 30-s all-out free-style swimming periods alternating with by 2 min of recovery) swim-training group, to the low-intensity continuous (60 min free-style) swimming group and the non-exercising control group [46,47]. Participants exercised 3 times a week for 15 weeks under supervision. Intermittent but not continuous swimming led to an improvement in insulin sensitivity (22%) and biomarkers of vascular function [47]. Both low volume (as little as 3–5 min per session) high-intensity interval and prolonged moderate-intensity swim training reduced systolic blood pressure, improved exercise capacity, reduced total body fat percentage, total fat mass, and body mass [46].

## 5. Be Eccentric

Muscle activity in relation to an external force is typically termed concentric when muscle activity is stronger than the external force, isometric when muscle activity equals the external force, or eccentric when muscle activity is lower than the external force. During eccentric work muscles lengthen and do negative work, such as lowering a load or slowing down a movement [48]. Examples for eccentric action in daily life include taking down heavy object from a height, or walking downhill or down the stairs. Eccentric muscle work is characterized by low energy cost and high force production [49]. Eccentric strength is preserved more during the aging process than concentric and isometric strength [50]. Low energy cost and high force production would qualify eccentric muscle activity as the optimal training modality for people with impaired muscular and cardiovascular fitness, such as the elderly and chronically ill, as well as the generally deconditioned [49]. Eccentric training has been however also associated with muscle and tendon damage [51]. More recent studies suggest that this is not inevitable, and the key in avoiding muscle damage, soreness and injury might be the dose, i.e., the magnitude and duration of the force production, which in turn is dependent on the load and the time in which eccentric work takes place [48,49]. In other words, eccentric exercise can be performed safely if the loads and duration of eccentric phases are selected appropriately and increased gradually [48,49,52]. Eccentric endurance and resistance exercise can improve functional capacity, body composition, enhancing muscle strength in the elderly [52]. The rating of perceived exertion (RPE) during eccentric exercise tends to be lower than during concentric activity, and it does not increase with increasing training intensity, which might contribute to high adherence [49,52].

A practical challenge in performing eccentric training is how to avoid the concentric portion, such as lifting weights, since special training machines are not widely available beyond study settings [52]. In a “real life” 4-month randomized crossover investigation, middle-aged sedentary participants were assigned either in the uphill (concentric) or the downhill (eccentric) hiking group (length 2.9 km, elevation difference 540 m) in the Austrian Alps [53,54]. For the respective opposite direction participants used a cable car free of charge. Participants were instructed to hike 3–5 times a week, which they then performed on their own, a compliance rate of a minimum of 3 times was achieved by 93% of the participants [53]. In line with previous studies energy expenditure per exercise bout was considerably higher in the uphill (442 ± 78 kcal) than in the downhill (127 ± 22 kcal) condition. Eccentric (downhill) hiking was found to be more “effective” (calculated as per kcal expended) at improving metabolic and inflammatory parameters. Improvements were about 3 and 4-fold higher in glucose tolerance, fasting low density lipoprotein cholesterol, and in homeostasis model assessment (HOMA) index of insulin resistance, serum fasting insulin, C-reactive protein respectively [53]. In hilly areas similar training might be feasible using public transportation to reach a higher point, from which descending is possible. 

A further pragmatic way of performing eccentric exercise is descending stairs (and taking the elevator to avoid concentric work). Chen and colleagues randomized sedentary elderly overweight women to perform either descending stair walking (DSW) or ascending stair walking (ASW) twice a week for 12 weeks [55]. An elevator was used for the respective opposite directions. Training volume per session increased gradually from about 5 min in week 1 to about 60 min in week 12. The average HR during stair walking was approximately 25 beats per minute lower for DSW than ASW, resting HR (−9.8% ± 4.3% vs. −4.0% ± 3.7%,) and systolic blood pressure (−8.6% ± 5.5% vs. −3.4% ± 3.4%) decreased more following DSW than ASW. An increase in bone mineral density was observed only for DSW (6.1% ± 5.2%). A more pronounced improvement in all markers of insulin sensitivity and blood lipid profile was found after DSW than ASW. Knee extensor muscle strength, some of the physical function outcomes improved more after DSW than ASW. RPE was lower in DSW, and no muscle soreness was reported in either group [55].

## 6. Pump It up No Iron Needed

Muscle mass, strength and quality play a crucial role in maintaining independence and enhancing cardiometabolic and musculoskeletal health throughout the average lifetime, as well as for older individuals. Accordingly, current health-oriented PA guidelines for adults recommend not only endurance but also muscle strengthening activities [6,15]. However, in public perception as well as in scientific publications, the focus tends to be on endurance-based activities. This is reflected in the fact that compliance or non-compliance with guidelines is often equated with performing or failing to perform 150 min of moderate (or 75 min of vigorous) intensity endurance type activity, with a relative neglect of muscle strengthening activities [8]. There are probably many reasons for this. Whereas the dose of 150 min precisely quantifies endurance activities, the specification for muscle strengthening activities is less clear cut, and the assessment of endurance type of activities is more straightforward. Some authors also suggest that traditional resistance training protocols might seem “complicated, time-consuming, and difficult” for the general population [56], which leads to low level of engagement in such activities [57]. While activities of daily life, such as walking and to a lesser extent cycling, are being promoted as being easily available health enhancing activities [58], pragmatic forms of muscle strengthening activities are also needed.

Because elastic bands are transportable and low cost, this type of resistance training can be an alternative to machine or free weights-based work out. A recent systematic review found that training with elastic bands improved muscle strength in “healthy” and “not healthy” older adults, with more pronounced effects in healthy participants [59]. Elastic resistance exercise also improves functional performance and muscle strength in adults in comparison with no exercise, and is equally effective in improving muscle strength, but not function as traditional resistance training [60]. Training with elastic bands and tubes can also improve body composition in overweight older [61] and postmenopausal women [62], and cardiometabolic markers such as C-reactive protein, glycosylated hemoglobin in patients with the metabolic syndrome [63].

A recent work place-based intervention randomized healthy employees with neck and shoulder pain to a 2 and 12 min exercise group and to the health information control group [64]. Participants in the exercise groups performed lateral raises using elastic tubes 5 times a week for 2 and 12 min respectively. The 10-week intervention resulted in clinically relevant reductions of pain and tenderness and increased muscle strength (maximal voluntary shoulder abduction at a static 90° shoulder joint angle), with no difference between the exercising groups [64]. In other words, as little as 2 min of daily progressive resistance training for 10 weeks was sufficient to achieve these results [64].

A further method to perform muscle strengthening activities is by using one’s own body weight. In an impressively simple 12-week randomized intervention patients with non-alcoholic fatty liver disease performed modified push-ups with knees on the floor and squats 3 times a week (3 sets of 10 push-ups and 3 sets of 10 squats, with 1 min rests between sets, for a total duration of 20–30 min) [65]. Participants were instructed to perform the eccentric phase slowly, hold for 1–2 s and do the concentric phase as fast as possible. This uncomplicated exercise intervention improved fat-free mass and muscle mass, hepatic steatosis grade, mean insulin and ferritin levels, and the homeostasis model assessment-estimated insulin resistance index [65].

A 5-week whole-body aerobic resistance training circuit using only body weight exercises increased aerobic fitness and muscular strength for the chest and hamstring muscles in sedentary young women [66]. Circuit training is becoming popular and has been shown to increase maximum oxygen uptake and one-repetition maximum bench presses in healthy adults [67]. Of the 16 studies included in this systematic review and meta-analysis only three were conducted in sedentary samples, and all three used training equipment. These data suggest that more studies using only body weight exercises in the general adult and older adult populations are warranted.

## 7. Discussion

Persistently low levels of PA in the general populations call for continued efforts in PA promotion. Providing evidence-based examples of forms of PA that do not require specific skills or equipment and are easily accessible to most, even novices and currently inactive individuals, might contribute to these efforts. As we show, there are a number of different ways of becoming physically active for the currently inactive.

All the examples above involve PA which is free of charge or of (very) low cost, and do not necessarily require memberships or high investments in gear or PA devices. Since low socioeconomic populations tend to be less active than higher ones, affordability might contribute to reducing health inequality. Furthermore, the volume of PA in all studies (in the non-comparator groups) was below the recommended level of 150 min per week. In the debate about the minimum amount of activity needed for meaningful health benefits, the focus so far has been on observational data. We now show a number of different experimental studies (RCT), with different interventions and forms of activity that corroborate observational studies. Since lack of time is one of the major barriers reported for not performing PA, evidence showing that even as little as 30 min of activity per week can improve health might help the inactive to adopt a more active lifestyle. Finally, most studies cited here employed PA forms that require no special skills or experience.

This is not a systematic review and the examples we quote here do not by any means question the validity of the general recommendations. Wherever possible, we tried to identify systematic reviews and meta-analyses to underpin our point, which was not always possible. In these cases, we cite randomized controlled trials (RCT), the second level of evidence after systematic reviews. A drawback of RCT however must be mentioned; because of the tight level of control of the intervention, generalizability of results might be limited. However, all studies mentioned here were conducted on non-athletes, typically middle-aged and older inactive adults, and as such, we are confident that results could be replicated in other studies.

## 8. Conclusions

We provide interventional evidence for the health enhancing effects of many easy to implement forms of PA feasible for the currently inactive. These examples also reinforce observational evidence on the beneficial effects of PA at lower doses than 150 min per week.

Many authors now agree that the most important PA-related public health goal should be increasing activity in those who currently engage in no activity at all. Accordingly, research efforts should be increased in identifying further forms of low volume, easily adoptable, low burden PA and strengthening the evidence base for those we present here, as well as in conducting effectiveness studies.

## References

[1] Kahlmeier S., Wijnhoven T.M.A., Alpiger P., Schweizer C., Breda J., Martin B.W. (2015). National physical activity recommendations: Systematic overview and analysis of the situation in European countries. BMC Public Health.

[2] Brawley L.R., Latimer A.E. (2007). Physical activity guides for Canadians: Messaging strategies, realistic expectations for change, and evaluation. Can. J. Public Health.

[3] Wen C.P., Wai J.P.M., Tsai M.K., Yang Y.C., Cheng T.Y.D., Lee M.-C., Chan H.T., Tsao C.K., Tsai S.P., Wu X. (2011). Minimum amount of physical activity for reduced mortality and extended life expectancy: A prospective cohort study. Lancet.

[4] Hupin D., Roche F., Gremeaux V., Chatard J.-C., Oriol M., Gaspoz J.-M., Barthélémy J.-C., Edouard P. (2015). Even a low-dose of moderate-to-vigorous physical activity reduces mortality by 22% in adults aged ≥60 years: A systematic review and meta-analysis. Br. J. Sports Med..

[5] Arem H., Moore S.C., Patel A., Hartge P., de Gonzalez A.B., Visvanathan K., Campbell P.T., Freedman M., Weiderpass E., Adami H.O. (2015). Leisure time physical activity and mortality: A detailed pooled analysis of the dose-response relationship. JAMA Int. Med..

[6] Physical Activity Guidelines Advisory Committee (2008). Physical Activity Guidelines Advisory Committee Report. https://health.gov/paguidelines/report/pdf/CommitteeReport.pdf.

[7] De Souto Barreto P. (2015). Global health agenda on non-communicable diseases: Has WHO set a smart goal for physical activity?. BMJ.

[8] Warburton D.E.R., Bredin S.S.D. (2016). Reflections on Physical Activity and Health: What Should We Recommend?. Can. J. Cardiol..

[9] Weed M. (2016). Evidence for physical activity guidelines as a public health intervention: Efficacy, effectiveness, and harm—A critical policy sciences approach. Health Psychol. Behav. Med..

[10] Hupin D., Edouard P., Gremeaux V., Roche F., Barthélémy J.-C. (2016). We need clear health messages about exercise. BMJ.

[11] De Souto Barreto P. (2015). Time to challenge public health guidelines on physical activity. Sports Med..

[12] Miyachi M., Tripette J., Kawakami R., Murakami H. (2015). “+10 min of Physical Activity per Day”: Japan Is Looking for Efficient but Feasible Recommendations for Its Population. J. Nutr. Sci. Vitaminol..

[13] Franco M.R., Tong A., Howard K., Sherrington C., Ferreira P.H., Pinto R.Z., Ferreira M.L. (2015). Older people’s perspectives on participation in physical activity: A systematic review and thematic synthesis of qualitative literature. Br. J. Sports Med..

[14] Cerin E., Leslie E., Sugiyama T., Owen N. (2010). Perceived barriers to leisure-time physical activity in adults: An ecological perspective. J. Phys. Act. Health.

[15] World Health Organisation (2010). Global Recommendations on Physical Activity for Health.

[16] Lee I.-M., Shiroma E.J. (2014). Using accelerometers to measure physical activity in large-scale epidemiological studies: Issues and challenges. Br. J. Sports Med..

[17] Shephard R.J. (2003). Limits to the measurement of habitual physical activity by questionnaires. Br. J. Sports Med..

[18] Füzéki E., Engeroff T., Banzer W. (2017). Health Benefits of Light-Intensity Physical Activity: A Systematic Review of Accelerometer Data of the National Health and Nutrition Examination Survey (NHANES). Sports Med..

[19] Huang G., Wang R., Chen P., Huang S.C., Donnelly J.E., Mehlferber J.P. (2016). Dose-response relationship of cardiorespiratory fitness adaptation to controlled endurance training in sedentary older adults. Eur. J. Prev. Cardiol..

[20] Nygaard H., Tomten S.E., Høstmark A.T. (2009). Slow postmeal walking reduces postprandial glycemia in middle-aged women. Appl. Physiol. Nutr. Metab..

[21] Lunde M.S.H., Hjellset V.T., Høstmark A.T. (2012). Slow post meal walking reduces the blood glucose response: An exploratory study in female Pakistani immigrants. J. Immigr. Minor. Health.

[22] Duncan J.J., Gordon N.F., Scott C.B. (1991). Women walking for health and fitness. How much is enough?. JAMA.

[23] Herzig K.-H., Ahola R., Leppäluoto J., Jokelainen J., Jämsä T., Keinänen-Kiukaanniemi S. (2014). Light physical activity determined by a motion sensor decreases insulin resistance, improves lipid homeostasis and reduces visceral fat in high-risk subjects: PreDiabEx study RCT. Int. J. Obes..

[24] Garber C.E., Blissmer B., Deschenes M.R., Franklin B.A., Lamonte M.J., Lee I.-M., Nieman D.C., Swain D.P. (2011). American College of Sports Medicine position stand. Quantity and quality of exercise for developing and maintaining cardiorespiratory, musculoskeletal, and neuromotor fitness in apparently healthy adults: Guidance for prescribing exercise. Med. Sci. Sports Exerc..

[25] Csapo R., Alegre L.M. (2016). Effects of resistance training with moderate vs. heavy loads on muscle mass and strength in the elderly: A meta-analysis. Scand J. Med. Sci. Sports.

[26] Watanabe Y., Madarame H., Ogasawara R., Nakazato K., Ishii N. (2014). Effect of very low-intensity resistance training with slow movement on muscle size and strength in healthy older adults. Clin. Physiol. Funct. Imaging.

[27] Tanimoto M., Ishii N. (2006). Effects of low-intensity resistance exercise with slow movement and tonic force generation on muscular function in young men. J. Appl. Physiol..

[28] Gearhart R.F., Goss F.L., Lagally K.M., Jakicic J.M., Gallagher J., Gallagher K.I., Robertson R.J. (2002). Ratings of perceived exertion in active muscle during high-intensity and low-intensity resistance exercise. J. Strength Cond. Res..

[29] Perri M.G., Anton S.D., Durning P.E., Ketterson T.U., Sydeman S.J., Berlant N.E., Kanasky W.F., Newton R.L., Limacher M.C., Martin A.D. (2002). Adherence to exercise prescriptions: Effects of prescribing moderate versus higher levels of intensity and frequency. Health Psychol. Off. J. Div. Health Psychol. Am. Psychol. Assoc..

[30] Sparling P.B., Howard B.J., Dunstan D.W., Owen N. (2015). Recommendations for physical activity in older adults. BMJ.

[31] Teh K.C., Aziz A.R. (2002). Heart rate, oxygen uptake, and energy cost of ascending and descending the stairs. Med. Sci. Sports Exerc..

[32] Boreham C.A., Wallace W.F., Nevill A. (2000). Training effects of accumulated daily stair-climbing exercise in previously sedentary young women. Prev. Med..

[33] Andersen L.L., Sundstrup E., Boysen M., Jakobsen M.D., Mortensen O.S., Persson R. (2013). Cardiovascular health effects of internet-based encouragements to do daily workplace stair-walks: Randomized controlled trial. J. Med. Int. Res..

[34] Bean J., Herman S., Kiely D.K., Callahan D., Mizer K., Frontera W.R., Fielding R.A. (2002). Weighted stair climbing in mobility-limited older people: A pilot study. J. Am. Geriatr. Soc..

[35] Hannan A.L., Hing W., Simas V., Climstein M., Coombes J.S., Jayasinghe R., Byrnes J., Furness J. (2018). High-intensity interval training versus moderate-intensity continuous training within cardiac rehabilitation: A systematic review and meta-analysis. Open Access J. Sports Med..

[36] Batacan R.B., Duncan M.J., Dalbo V.J., Tucker P.S., Fenning A.S. (2017). Effects of high-intensity interval training on cardiometabolic health: A systematic review and meta-analysis of intervention studies. Br. J. Sports Med..

[37] Ribeiro P.A.B., Boidin M., Juneau M., Nigam A., Gayda M. (2017). High-intensity interval training in patients with coronary heart disease: Prescription models and perspectives. Ann. Phys. Rehabil. Med..

[38] Wormgoor S.G., Dalleck L.C., Zinn C., Harris N.K. (2017). Effects of High-Intensity Interval Training on People Living with Type 2 Diabetes: A Narrative Review. Can. J. Diabetes.

[39] Nemoto K.-I., Gen-no H., Masuki S., Okazaki K., Nose H. (2007). Effects of high-intensity interval walking training on physical fitness and blood pressure in middle-aged and older people. Mayo Clin. Proc..

[40] Masuki S., Mori M., Tabara Y., Sakurai A., Hashimoto S., Morikawa M., Miyagawa K., Sumiyoshi E., Miki T., Higuchi K. (2015). The factors affecting adherence to a long-term interval walking training program in middle-aged and older people. J. Appl. Physiol..

[41] Nose H., Morikawa M., Yamazaki T., Nemoto K.-I., Okazaki K., Masuki S., Kamijo Y.-I., Gen-no H. (2009). Beyond epidemiology: Field studies and the physiology laboratory as the whole world. J. Physiol..

[42] Lalande S., Okazaki K., Yamazaki T., Nose H., Joyner M.J., Johnson B.D. (2010). Effects of interval walking on physical fitness in middle-aged individuals. J. Prim. Care Community Health.

[43] Karstoft K., Winding K., Knudsen S.H., Nielsen J.S., Thomsen C., Pedersen B.K., Solomon T.P.J. (2013). The effects of free-living interval-walking training on glycemic control, body composition, and physical fitness in type 2 diabetic patients: A randomized, controlled trial. Diabetes Care.

[44] Brinkløv C.F., Thorsen I.K., Karstoft K., Brøns C., Valentiner L., Langberg H., Vaag A.A., Nielsen J.S., Pedersen B.K., Ried-Larsen M. (2016). Criterion validity and reliability of a smartphone delivered sub-maximal fitness test for people with type 2 diabetes. BMC Sports Sci. Med. Rehabilit..

[45] Ried-Larsen M., Thomsen R.W., Berencsi K., Brinkløv C.F., Brøns C., Valentiner L.S., Karstoft K., Langberg H., Vaag A.A., Pedersen B.K. (2016). Implementation of interval walking training in patients with type 2 diabetes in Denmark: Rationale, design, and baseline characteristics. Clin. Epidemiol..

[46] Mohr M., Nordsborg N.B., Lindenskov A., Steinholm H., Nielsen H.P., Mortensen J., Weihe P., Krustrup P. (2014). High-intensity intermittent swimming improves cardiovascular health status for women with mild hypertension. BioMed Res. Int..

[47] Connolly L.J., Nordsborg N.B., Nyberg M., Weihe P., Krustrup P., Mohr M. (2016). Low-volume high-intensity swim training is superior to high-volume low-intensity training in relation to insulin sensitivity and glucose control in inactive middle-aged women. Eur. J. Appl. Physiol..

[48] Hoppeler H. (2016). Moderate Load Eccentric Exercise; A Distinct Novel Training Modality. Front. Physiol..

[49] LaStayo P., Marcus R., Dibble L., Frajacomo F., Lindstedt S. (2014). Eccentric exercise in rehabilitation: Safety, feasibility, and application. J. Appl. Physiol..

[50] Vandervoort A.A. (2002). Aging of the human neuromuscular system. Muscle Nerve.

[51] Proske U., Morgan D.L. (2001). Muscle damage from eccentric exercise: Mechanism, mechanical signs, adaptation and clinical applications. J. Physiol..

[52] Gluchowski A., Harris N., Dulson D., Cronin J. (2015). Chronic Eccentric Exercise and the Older Adult. Sports Med..

[53] Zeppetzauer M., Drexel H., Vonbank A., Rein P., Aczel S., Saely C.H. (2013). Eccentric endurance exercise economically improves metabolic and inflammatory risk factors. Eur. J. Prev. Cardiol..

[54] Drexel H., Saely C.H., Langer P., Loruenser G., Marte T., Risch L., Hoefle G., Aczel S. (2008). Metabolic and anti-inflammatory benefits of eccentric endurance exercise—A pilot study. Eur. J. Clin. Investig..

[55] Chen T.C., Hsieh C.-C., Tseng K.-W., Ho C.-C., Nosaka K. (2017). Effects of Descending Stair Walking on Health and Fitness of Elderly Obese Women. Med. Sci. Sports Exerc..

[56] Phillips S.M., Winett R.A. (2010). Uncomplicated resistance training and health-related outcomes: Evidence for a public health mandate. Curr. Sports Med. Rep..

[57] Katzmarzyk P.T., Lee I.-M., Martin C.K., Blair S.N. (2017). Epidemiology of Physical Activity and Exercise Training in the United States. Prog. Cardiovasc. Dis..

[58] Lee I.-M., Buchner D.M. (2008). The importance of walking to public health. Med. Sci. Sports Exerc..

[59] Martins W.R., de Oliveira R.J., Carvalho R.S., de Oliveira Damasceno V., da Silva V.Z.M., Silva M.S. (2013). Elastic resistance training to increase muscle strength in elderly: A systematic review with meta-analysis. Arch. Gerontol. Geriatr..

[60] De Oliveira P.A., Blasczyk J.C., Souza Junior G., Lagoa K.F., Soares M., de Oliveira R.J., Filho P.J.B.G., Carregaro R.L., Martins W.R. (2017). Effects of Elastic Resistance Exercise on Muscle Strength and Functional Performance in Healthy Adults: A Systematic Review and Meta-Analysis. J. Phys. Act. Health.

[61] Fritz N.B., Juesas Á., Gargallo P., Calatayud J., Fernández-Garrido J., Rogers M.E., Colado J.C. (2018). Positive Effects of a Short-Term Intense Elastic Resistance Training Program on Body Composition and Physical Functioning in Overweight Older Women. Biol. Res. Nurs..

[62] Colado J.C., Garcia-Masso X., Rogers M.E., Tella V., Benavent J., Dantas E.H. (2012). Effects of aquatic and dry land resistance training devices on body composition and physical capacity in postmenopausal women. J. Hum. Kinet..

[63] Flandez J., Belando N., Gargallo P., Fernández-Garrido J., Vargas-Foitzick R.A., Devis-Devis J., Colado J.C. (2017). Metabolic and Functional Profile of Premenopausal Women with Metabolic Syndrome after Training with Elastics as Compared to Free Weights. Biol. Res. Nurs..

[64] Andersen L.L., Saervoll C.A., Mortensen O.S., Poulsen O.M., Hannerz H., Zebis M.K. (2011). Effectiveness of small daily amounts of progressive resistance training for frequent neck/shoulder pain: Randomised controlled trial. Pain.

[65] Takahashi A., Abe K., Usami K., Imaizumi H., Hayashi M., Okai K., Kanno Y., Tanji N., Watanabe H., Ohira H. (2015). Simple Resistance Exercise helps Patients with Non-alcoholic Fatty Liver Disease. Int. J. Sports Med..

[66] Myers T.R., Schneider M.G., Schmale M.S., Hazell T.J. (2015). Whole-body aerobic resistance training circuit improves aerobic fitness and muscle strength in sedentary young females. J. Strength Cond. Res..

[67] Muñoz-Martínez F.A., Rubio-Arias J.Á., Ramos-Campo D.J., Alcaraz P.E. (2017). Effectiveness of Resistance Circuit-Based Training for Maximum Oxygen Uptake and Upper-Body One-Repetition Maximum Improvements: A Systematic Review and Meta-Analysis. Sports Med..

